# Absence of renal hypoxia in the subacute phase of severe renal ischemia-reperfusion injury

**DOI:** 10.1152/ajprenal.00249.2018

**Published:** 2018-08-15

**Authors:** Connie P. C. Ow, Jennifer P. Ngo, Md Mahbub Ullah, Giannie Barsha, Ruth C. Meex, Matthew J. Watt, Lucinda M. Hilliard, Maarten P. Koeners, Roger G. Evans

**Affiliations:** ^1^Cardiovascular Disease Program, Biomedicine Discovery Institute, Department of Physiology, Monash University, Melbourne, Victoria, Australia; ^2^Department of Human Biology, NUTRIM School of Nutritional and Translational Research in Metabolism, Maastricht University Medical Centre, Maastricht, The Netherlands; ^3^Metabolism, Diabetes and Obesity Program, Biomedicine Discovery Institute, Department of Physiology, Monash University, Melbourne, Victoria, Australia; ^4^School of Physiology, Pharmacology and Neuroscience, Biomedical Sciences, University of Bristol, Bristol, United Kingdom; ^5^Institute of Biomedical and Clinical Science, University of Exeter Medical School, Exeter, United Kingdom

**Keywords:** acute kidney injury, hypoxia, ischemia-reperfusion, kidney, oxygen

## Abstract

Tissue hypoxia has been proposed as an important event in renal ischemia-reperfusion injury (IRI), particularly during the period of ischemia and in the immediate hours following reperfusion. However, little is known about renal oxygenation during the subacute phase of IRI. We employed four different methods to assess the temporal and spatial changes in tissue oxygenation during the subacute phase (24 h and 5 days after reperfusion) of a severe form of renal IRI in rats. We hypothesized that the kidney is hypoxic 24 h and 5 days after an hour of bilateral renal ischemia, driven by a disturbed balance between renal oxygen delivery (Do_2_) and oxygen consumption (V̇o_2_). Renal Do_2_ was not significantly reduced in the subacute phase of IRI. In contrast, renal V̇o_2_ was 55% less 24 h after reperfusion and 49% less 5 days after reperfusion than after sham ischemia. Inner medullary tissue Po_2_, measured by radiotelemetry, was 25 ± 12% (mean ± SE) greater 24 h after ischemia than after sham ischemia. By 5 days after reperfusion, tissue Po_2_ was similar to that in rats subjected to sham ischemia. Tissue Po_2_ measured by Clark electrode was consistently greater 24 h, but not 5 days, after ischemia than after sham ischemia. Cellular hypoxia, assessed by pimonidazole adduct immunohistochemistry, was largely absent at both time points, and tissue levels of hypoxia-inducible factors were downregulated following renal ischemia. Thus, in this model of severe IRI, tissue hypoxia does not appear to be an obligatory event during the subacute phase, likely because of the markedly reduced oxygen consumption.

## INTRODUCTION

Acute kidney injury (AKI) is a major cause of death and disability globally and places a major acute burden on health care systems (26). It also renders patients more susceptible to later development of chronic kidney disease (CKD; 2). For example, a diagnosis of AKI was found to be associated with an 8.8-fold excess risk of later development of CKD (8). Furthermore, the risk of later development of CKD increases with the severity of AKI (8). Tissue hypoxia has been proposed as an important driver in the pathogenesis of both AKI and CKD, although this proposition remains to be definitively tested (33).

Ischemia-reperfusion injury (IRI) sustained from medical interventions often arises from the obligatory need to restrict or completely prevent blood flow to the kidney, resulting in a period of severe hypoxia or complete anoxia (15). Cellular damage such as acute tubular necrosis and tubular apoptosis is evident during the reperfusion period and is likely driven in part by the presence of tissue hypoxia during the period of ischemia. In experimental IRI, cortical (27, 28, 41) and medullary (27, 28, 34) tissue hypoxia has also been observed during the first few hours of reperfusion after complete renal ischemia. Importantly, in the longer term (weeks to months) after renal IRI the kidney was observed to be hypoxic even with some level of, albeit incomplete, structural and functional recovery (3, 4). However, there are few available data regarding renal tissue oxygenation beyond the first few hours of reperfusion during the extension and recovery phases of IRI. This information is required if we are to understand the role of tissue hypoxia in the natural history of AKI, either as it progresses to end-stage renal disease or as renal function recovers but the risk of later CKD is increased.

The chief aim of the present study was to assess the time course of changes in, and the spatial distribution of, tissue oxygen tension (Po_2_) during the subacute phase of severe IRI (the first 5 days of reperfusion after 60 min of bilateral renal ischemia). We chose severe IRI in an attempt to model the clinical situation of severe AKI leading to end-stage renal disease, cognizant of the possibility that renal oxygenation in this scenario might differ considerably from that in milder forms of renal IRI. We tested the hypothesis that renal tissue is hypoxic during the subacute phase of IRI. Four approaches were used for assessment of renal tissue oxygenation, each with varying temporal and spatial resolution. Radiotelemetry was used to examine the time course of changes in inner medullary tissue Po_2_ in freely moving rats (22, 23). Clark-type electrodes were used to characterize the spatial variations in renal tissue Po_2_ in the renal cortex and medulla of anesthetized rats at both 24 h and 5 days after reperfusion. This experiment also provided an opportunity to determine the contribution of changes in renal oxygen delivery (Do_2_) and oxygen consumption (V̇o_2_) to alterations in renal tissue Po_2_ 24 h and 5 days after reperfusion. Pimonidazole adduct immunohistochemistry was used to characterize the spatial distribution of cellular hypoxia 24 h and 5 days after reperfusion. We also measured the expression of hypoxia-inducible factors (HIF-1α and HIF-2α) and some of their downstream gene targets.

## METHODS

### Experimental Animals

Ten- to twelve-week-old male, Sprague-Dawley rats (*n* = 70) were obtained from the Animal Resources Centre (Perth, WA, Australia). They were housed in a room maintained at 21–23°C with a 12-h light-dark cycle. The rats were allowed free access to water and standard laboratory rat chow. All procedures were approved in advance by the Animal Ethics Committee of the School of Biomedical Sciences, Monash University, as being in accordance with the Australian Code of Practice for the Care and Use of Animals for Scientific Purposes.

### Induction of Bilateral Renal Ischemia

Rats were anesthetized with isoflurane (IsoFlo, 05260-05; Abbott Laboratories, Abbott Park, IL), using a vaporizer, and maintained at 2.5–3.0% vol/vol. A midline incision was made to expose the left and right renal arteries. To induce bilateral renal ischemia (*n* = 36), blood flow to both kidneys was prevented by the application of microvascular clamps (no. 00398; S&T, Neuhausen am Rheinfall, Switzerland) placed on both the left and right renal arteries and veins. Complete ischemia was confirmed by observing the blanching of the kidneys. After an hour, the microvascular clamps were removed, so blood flow to both kidneys was restored. Wounds were closed in layers with sutures, and each rat was then allowed to recover from the surgery on a heated pad for an hour. A separate cohort of rats (*n* = 34) underwent the same procedure with the exception of the application of the microvascular clamps and so served as controls (sham ischemia). Rats received subcutaneous injections of an analgesic (carprofen, 1.25 mg; Pfizer) for 2 consecutive days following recovery from surgery.

### Protocol 1: Temporal Changes in Renal Tissue Oxygenation Following Renal Ischemia

We employed a radiotelemetric method (22, 23) to characterize the temporal profile of changes in renal tissue Po_2_ after renal ischemia and reperfusion. Briefly, the oxygen telemeter was implanted under isoflurane anesthesia so that the tip of the oxygen-sensing carbon paste electrode was in the inner medulla of the left kidney (5 mm below the renal capsule). One week after implantation of the telemetric probe, the rats underwent a second surgical procedure for the induction of either bilateral renal ischemia (*n* = 7, body weight = 501 ± 20 g, mean ± SE) or sham ischemia (*n* = 5, body weight = 491 ± 21 g). Renal tissue Po_2_ was recorded continuously for 1 day before and for 5 days after recovery from surgery. Rats received subcutaneous injections of an analgesic (carprofen, 1.25 mg; Pfizer, Australia) before laparotomy and for 2 consecutive days following recovery from surgery.

#### Measurements and calculations.

Current measured by the telemeters was filtered with a 25-Hz low-pass filter, and artifactual measurements were removed when the first-order derivative of the measured current exceeded the threshold of 5–500 nA/s. The zero-offset current, acquired when the rat was killed at the end of the study via induction of cardiac arrest under anesthesia (22), was determined and subtracted. Data are presented as a percentage of the average value on the day before surgery to induce ischemia or sham ischemia.

### Protocol 2: Renal Tissue Oxygenation and Its Determinants After Renal Ischemia

Either 24 h or 5 days following recovery from renal ischemia or sham ischemia, rats were anesthetized and prepared for the assessment of regional tissue Po_2_ using a Clark electrode (50-μm tip, OX-50; Unisense, Aarhus, Denmark). We assessed *1*) cortical tissue Po_2_ across a range of sites on the dorsal surface of the kidney and *2*) a profile of tissue Po_2_ with depth from the cortical surface. In this set of studies, we also determined the major determinants of tissue Po_2_, renal Do_2_, and V̇o_2_.

Rats (*n* = 6–11 per group) were anesthetized with sodium thiobutabarbital (100 mg/kg ip, Inactin; Sigma-Aldrich, St. Louis, MO). A tracheostomy was performed to facilitate artificial ventilation with 40% inspired oxygen at a ventilation rate of 90–100 breaths/min and a tidal volume of 3.5 ml (Ugo Basile, model 7025; SDR Clinical Technology, Sydney, NSW, Australia) as previously described (1). The left carotid artery was catheterized to facilitate arterial blood sampling and blood pressure measurement. The right jugular vein was catheterized to facilitate infusion of maintenance fluid (154 mM NaCl) at a rate of 6 ml/h during the period of surgical preparation. The bladder was catheterized, for collection of urine from the left kidney, for assessment of renal function using standard clearance methods. The degree of saturation of hemoglobin with oxygen was measured continuously using a sensor placed on the foot (Mouse Ox; Starr Life Sciences, Oakmont, PA).

The right renal artery and vein were ligated, and a catheter was passed from the right renal vein through the vena cava and into the left renal vein for the sampling of renal venous blood. Total renal blood flow (RBF) was measured using a transit time ultrasound flow probe (type 0.7 VB; Transonic Systems, Ithaca, NY) placed around the left renal artery. Following completion of the surgical preparations, rats received bolus doses of [^3^H]inulin (10 μCi in 50 μl; PerkinElmer Australia, Melbourne, VIC, Australia) and pancuronium bromide (2 mg/kg; AstraZeneca, Sydney, NSW, Australia) intravenously. A maintenance infusion of 2% wt/vol bovine serum albumin (Sigma-Aldrich) in 154 mM sodium chloride delivered 676 nCi/h [^3^H]inulin and 0.1 mg·kg^−1^·h^−1^ pancuronium bromide through the jugular vein at a rate of 2 ml/h. The infusion commenced once all surgical preparations were completed and was maintained throughout the rest of the protocol.

After a 1-h equilibration period, a 0.5-ml sample of arterial blood was taken for blood oximetry. The plasma component of the sample was later used for assessment of the concentrations of [^3^H]inulin and sodium. A 0.1-ml sample of renal venous blood was also collected for blood oximetry. Renal tissue Po_2_ was then assessed using a Clark electrode attached to a micromanipulator. Two series of measurements were taken. In the first series, the electrode was advanced 2 mm from the renal surface, into the cortex, at six randomly chosen sites across the left kidney. The second series established a profile of tissue Po_2_ with depth below the cortical surface. The electrode was moved to the midpoint of the cortical surface of the kidney and advanced into the kidney at 1-mm increments up to a depth of 10 mm from the renal surface as previously described (32). Once all measurements were taken, a second set of blood samples, from the carotid artery and the renal vein, was taken as before. Urine made by the left kidney, during the period of measurement of tissue Po_2_, was collected for measurement of the concentrations of [^3^H]inulin and sodium.

#### Measurements and calculations.

Arterial pressure, heart rate (triggered by arterial pressure), RBF, core body and tissue temperature, and renal tissue Po_2_ measured by Clark electrode were digitized as previously described (32). Urinary and plasma concentrations of sodium were determined using ion-sensitive electrodes (EasyElectrolytes; Medica, Bedford, MA). Glomerular filtration rate (GFR) was determined by the clearance of [^3^H]inulin. Blood chemistry was assessed using a point-of-care device (iSTAT, CG8+ cartridges; Abbott Laboratories). Arterial and venous blood oxygen content was calculated as previously described (1).

### Protocol 3: Cellular Hypoxia and Hypoxic Signaling After Renal Ischemia

Either after 24 h or 5 days of recovery from bilateral renal ischemia or sham ischemia (*n* = 6 per group), rats were prepared for perfusion fixation of the right kidney. In this set of studies, the chief aim was to assess cellular hypoxia using pimonidazole adduct immunohistochemistry. Pimonidazole chloride (HP1-1000 kit; Hydroxyprobe) was administered, at a dose of 60 mg/kg ip 3 h before perfusion fixation of the kidney.

Three hours after the injection of pimonidazole, rats were anesthetized with sodium pentobarbital (60 mg/kg ip; Sigma-Aldrich). The left carotid artery was catheterized to facilitate arterial blood sampling. A midline incision was then made exposing both kidneys and the bladder. A urine sample was taken by puncturing the bladder wall and was frozen at −20°C for later analysis. The left renal artery and vein were isolated and freed from surrounding connective tissue and fat. Lidocaine (2% wt/vol, Xylocaine; AstraZeneca) was applied onto both vessels to prevent spasm of the renal artery. Silk ligatures (3-0 Dysilk; Dynek, Hendon, SA, Australia) were placed around the vena cava above the level of the right kidney, around the left renal artery and vein, and around the abdominal aorta. An incision was made in the abdominal aorta below the level of the left kidney, and a polyurethane catheter connected to the perfusion apparatus was advanced into the aorta, facing upstream, thereby facilitating retrograde perfusion. A 1-ml blood sample was taken from the carotid artery for later analysis. The left renal artery and vein were then ligated, and the left kidney was removed, decapsulated, and snap-frozen in liquid nitrogen for later analysis of HIF-1α and HIF-2α protein and gene expression of HIF-1α, HIF-2α, VEGF-α, and heme oxygenase 1 (HO-1). Prior to freezing, the left kidney was sectioned in the coronal plane into 4–5 slices of ~1–2-mm thickness.

The ligatures surrounding the vena cava and abdominal aorta were tied off, and the right kidney was perfused with 100–150 ml of 4% wt/vol paraformaldehyde (paraformaldehyde powder, no. 158127; Sigma-Aldrich) at room temperature and a pressure of 150 mmHg. The inferior vena cava was incised to vent perfusate. The perfused kidney was removed, decapsulated, and stored in 4% paraformaldehyde for 48 h before it was processed for embedding and staining at the Monash Histology Platform.

Blood chemistry was assessed using a point-of-care device (iSTAT, CHEM8+ cartridges; Abbott Laboratories). Urinary albumin concentration was determined using direct competitive enzyme-linked immunosorbent assay (Nephrat II, NR-002; Exocell, Philadelphia, PA). Urinary creatinine concentration was determined using an assay based on Jaffe’s reaction of alkaline picrate solution with creatinine (Creatinine Companion Strip Plate, no. 1012; Exocell).

#### Quantification of fibrosis.

The right kidney was processed, embedded in paraffin, and sectioned at a thickness of 5 μm in the coronal plane. Collagen deposition was assessed by staining with 1% wt/vol picrosirius red. The cortical and outer and inner medullary region of the kidney in each section was identified using Aperio ImageScope (Leica Biosystems Imaging). The amount of collagen deposited was quantified as a percentage of the entire area in each region.

#### Pimonidazole adduct immunohistochemistry.

Antigen retrieval was performed by incubating the sections in citrate buffer (Target Retrieval Solution; Dako) at 90°C for 30 min. Sections were then washed in Tris-buffered saline (154 mM NaCl) with Tween 20 (TBST; Dako) once they had cooled to 80°C. Excessive tissue peroxidase activity was then quenched using 0.03% vol/vol hydrogen peroxide containing sodium azide (Dako) for 10 min. Sections were then incubated in a protein block serum (Protein Block Serum-Free; Dako) for 10 min, to remove nonspecific binding, and washed twice more in TBST. Sections were then treated with an affinity-purified polyclonal anti-pimonidazole antibody raised in the rabbit (1:200 dilution, PAb2627AP; Hydroxyprobe) for 1 h at room temperature before incubation in goat anti-rabbit secondary antibody conjugated with horseradish peroxidase (HRP; polyclonal goat, EnVision; Dako) for 30 min at room temperature. Sections were washed twice with TBST before incubation with 3-diaminobenzidine (Dako) for 10 min and then counterstained with hematoxylin (Automation Hematoxylin; Dako) before coverslips were mounted.

#### Western blot analysis of HIF-1α and HIF-2α proteins.

The snap-frozen kidney was thawed, and the cortex and outer and inner medulla inclusive of the papilla were rapidly dissected. To stop further enzymatic reactions, the tissue samples were placed in radioimmunoprecipitation assay buffer [8 μl/mg; consisting of 50 mM Tris·HCl, 150 mM NaCl, 0.1% Triton X-100, 0.5% sodium deoxycholate, 0.1% sodium dodecyl sulfate (SDS), 1 mM sodium orthovanadate, 1 mM NaF, 1:25 of 25X phosphatase inhibitor, 1:10 of 10X PhosphoSTOP, and 1:1,000 dithiothreitol]. The tissues were then homogenized at 14,000 rpm at 4°C for 20 min, and equal amounts of protein (30 µg, determined by a Bradford protein assay) were loaded into a 7.5% precast gel (7.5% Mini-Protean TGX Precast Protein Gels, 4561025; Bio-Rad Laboratories) and fractionated electrophoretically in Tris-glycine-SDS running buffer at 300 V for 20 min. The fractionated protein in the gel was then transferred onto a nitrocellulose membrane (Bio-Rad Laboratories). Nonspecific binding was blocked with 5% skim milk in TBST buffer. As the primary antibodies for HIF-1α (NB100-479; Novus Biologicals, Littleton, CO) and HIF-2α (NB100-122; Novus Biologicals) are similar in molecular mass (115 and 118 kDa), we performed the immunoblot analysis of each protein of interest on separate gels. The nitrocellulose membranes were incubated overnight at 4°C in the primary antibody (1:1,000, raised in rabbit) made up in a solution of 2.5% wt/vol bovine serum albumin. The membranes were then incubated with 1:4,000 secondary antibody (ECL anti-rabbit IgG, HRP-linked whole antibody; GE Healthcare) and 1:15,000 conjugate (Precision Protein StrepTactin-HRP conjugate; Bio-Rad Laboratories) for an hour at room temperature. The nitrocellulose membrane was developed using equal parts of Clarity Western Peroxide Reagent (Bio-Rad Laboratories) and Clarity Western Luminol/Enhancer Reagent (Bio-Rad Laboratories) for 3 min before imaging. The intensity of the bands observed on the membrane was quantified and corrected for variability in protein migration down the gel and for total protein content loaded into the wells. Comparisons were made between treatment groups across the two time points within each region (i.e., cortex and outer and inner medulla).

#### Quantitative real-time PCR.

The tissue samples were homogenized, and total RNA was isolated using the RNeasy Mini Kit (no. 74104; Qiagen). Predesigned assays for primers of the 18s housekeeping gene (Rn03928990_g1) and HIF-1α (Rn01472831_m1), HIF-2α (Rn00576515_m1), VEGF-α (Rn01511602_m1), and HO-1 (Rn00561387_m1) genes were obtained from Thermo Fisher Scientific. Real-time PCR was performed on ABI 7900 HT (Thermo Fisher Scientific). Data were calculated by the 2^−ΔΔCt^ method (where Ct is threshold cycle).

### Statistical Analysis

Statistical analyses were performed using the software package SYSTAT (version 13; Systat Software, San Jose, CA). Two-sided *P* ≤ 0.05 was considered statistically significant. Normality was assessed using the Shapiro-Wilk test (40). Data that did not violate normality are presented as means ± SE, whereas data that violated normality are presented as medians (25th percentile, 75th percentile). Analysis of variance (ANOVA) was used to assess the independent effects of treatment and time and their interaction. For data that violated normality, an ANOVA on ranking (9) was performed instead. Dichotomous comparisons of continuous variables were made using Student’s *t*-test for data that did not violate normality, and the Mann-Whitney *U*-test was performed for data that violated normality. To protect against the risk of type I error arising from multiple comparisons, *P* values were conservatively adjusted using the Dunn-Sidak procedure (30). *P* values derived from within-subjects factors in repeated-measures ANOVA were conservatively adjusted using the Greenhouse-Geisser method (31).

## RESULTS

### Protocol 1: Temporal Changes in Renal Tissue Oxygenation Following Renal Ischemia

**Fig. 1.**
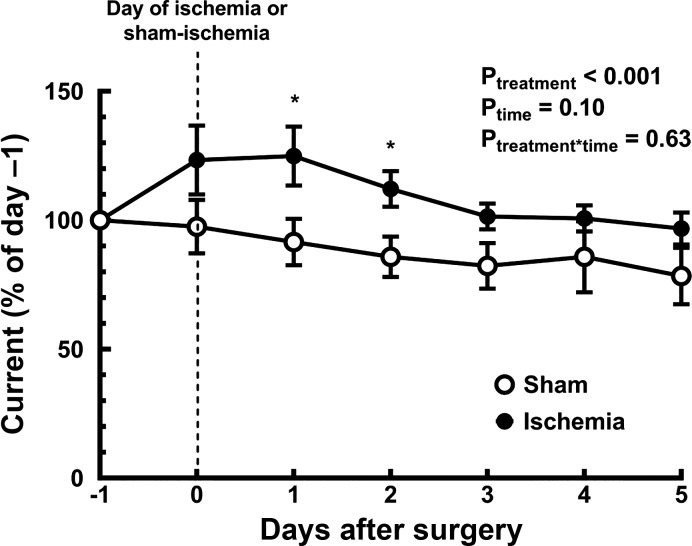
Temporal changes of inner medullary tissue Po_2_ following renal ischemia or sham ischemia. Values are means ± SE for rats subjected to either an hour of sham (*n* = 5) or bilateral renal ischemia (*n* = 7). Tissue Po_2_, assessed as current through the carbon paste electrode, was recorded before (*day −1*) and after (*days 0–5*) surgery. Current was averaged over each 24-h period and is expressed as a percentage of its mean value on the day before the surgery (*day −1*). *P*_treatment_, *P*_time_, and *P*_treatment*time_ are the outcomes of a two-way repeated-measures analysis of variance with factors treatment and time. *P ≤ 0.05 for specific comparisons between the two treatment groups at each time point using Student’s unpaired *t*-test, without correction for multiple comparisons.

On the first day after reperfusion, inner medullary tissue Po_2_ measured by telemetry was 25 ± 12% greater than its control level (*day −1*; Fig. 1). Tissue Po_2_ then gradually fell to be close to its control level by the fifth day after reperfusion of the kidney. After sham ischemia, inner medullary tissue Po_2_ tended to gradually fall and so was 22 ± 11% less than its control level by *day 5* after surgery.

### Protocol 2: Renal Tissue Oxygenation and Its Determinants After Renal Ischemia

#### Systemic parameters.

Twenty-four hours after reperfusion, body weight did not differ significantly from that of rats that underwent sham ischemia. By 5 days after renal ischemia, rats had lost 39.2 ± 6.1 g of their body weight. Left kidney weight 24 h after renal ischemia was similar to that after sham ischemia. In contrast, left kidney weight was 56% greater 5 days following renal ischemia than after sham ischemia (Table 1). Mean arterial pressure was similar in the two groups of rats at both 24 h and 5 days after surgery.

**Table 1.** Systemic and blood oxygen parameters of rats 24 h or 5 days after ischemia or sham ischemia

	Sham (24 h)		Ischemia (24 h)		Sham (5 days)		Ischemia (5 days)		2-Way ANOVA			Dichotomous Comparison	
Parameter	Value	*n*	Value	*n*	Value	*n*	Value	*n*	*P*_Tr_	*P*_T_	*P*_Tr*T_	S1 vs. I1	S5 vs. I5
Systemic													
Body weight after ischemia or sham ischemia, g	397.6 ± 23.8	10	378.2 ± 17.6	9	469.4 ± 14.9	7	364.5 ± 19.7	8	0.005	0.17	0.05	0.77	0.002
Kidney weight, g	1.5 (1.3, 1.5)	10	1.5 (1.4, 1.6)	9	1.4 (1.4, 1.7)	7	2.2 (2.2, 2.8)	8	<0.001	<0.001	0.04	0.92	<0.001
Kidney weight, g/kg body wt	3.6 (3.3, 4.1)	10	3.9 (3.7, 4.2)	9	3.3 (3.2, 3.5)	7	6.6 (5.4, 8.3)	8	<0.001	0.16	<0.001	0.55	0.002
Mean arterial blood pressure, mmHg	118.3 (108.4, 140.4)	10	123.9 (108.7, 134.4)	9	116.9 (102.3, 118.7)	7	116.3 (87.2, 122.3)	8	0.59	0.07	0.79	0.86	0.93
Blood oximetry													
Arterial blood Po_2_, mmHg	113.7 ± 6.1	10	89.2 ± 4.7	9	97.4 ± 5.0	7	100.7 ± 7.7	8	0.09	0.70	0.03	0.01	0.93
Hematocrit, %	44.8 (41.9, 45.6)	10	38.5 (35.4, 40.3)	9	43.0 (41, 44.5)	7	42.1 (41.4, 43.3)	8	0.01	0.35	0.06	0.04	0.07
Arterial blood oxygen saturation, %	98.3 (97.1, 99.0)	10	96.5 (92.3, 97.5)	9	96.5 (94, 97.5)	7	97.5 (96.6, 98.5)	8	0.36	0.80	0.003	0.02	0.22
Renal oxygen delivery, μmol/min	30.5 ± 2.7	10	21.7 ± 2.4	9	29.7 ± 5.5	7	20.8 ± 2.9	8	0.03	0.95	0.99	0.06	0.28
Renal oxygen delivery, nmol·min^−1^·g body wt^−1^	80.5 ± 2.7	10	57.7 ± 5.6	9	64.3 ± 12.7	7	56.5 ± 6.8	8	0.20	0.60	0.60	0.10	0.82
Renal oxygen consumption, μmol/min	2.9 (1.1, 6.0)	6	1.1 (0.7, 2.7)	7	2.6 (1.8, 4.1)	7	1.4 (0.9, 2.1)	8	0.04	0.99	0.99	0.30	0.06
Renal oxygen consumption, nmol·min^−1^·g body wt^−1^	8.3 ± 2.8	6	4.1 ± 1.1	7	6.1 ± 1.2	7	3.8 ± 0.6	8	0.08	0.70	0.80	0.30	0.20
Fractional extraction O_2_, %	10.1 (6.0, 17.8)	6	5.1 (3.3, 11.7)	7	7.9 (7.1, 14.4)	7	6.9 (4.7, 8.2)	8	0.11	0.95	0.70	0.44	0.51

Values of variables that did not violate normality are means ± SE, whereas values of variables that violated normality are medians (25th percentile, 75th percentile); *n* = no. of rats. Normality of the data was assessed using the Shapiro-Wilk test. *P*_Tr_, *P*_T_, and *P*_Tr*T_ are the outcomes of 2-way analysis of variance (ANOVA) with factors treatment (Tr) and time (T) for data that did not violate normality. For data that violated normality, an ANOVA on ranking was performed instead. Dichotomous comparisons of continuous variables were made using Student’s *t*-test for data that did not violate normality. For data that violated normality, a Mann-Whitney *U*-test was performed for dichotomous comparisons. *P* values for dichotomous comparisons were conservatively adjusted using the Dunn-Sidak correction with *k* = 2 to account for the fact that comparisons were made at 24 h and 5 days. I1, 24 h after ischemia and reperfusion; I5, 5 days after ischemia and reperfusion; S1, 24 h after sham ischemia; S5, 5 days after sham ischemia.

#### Renal tissue oxygenation.

**Fig. 2.**
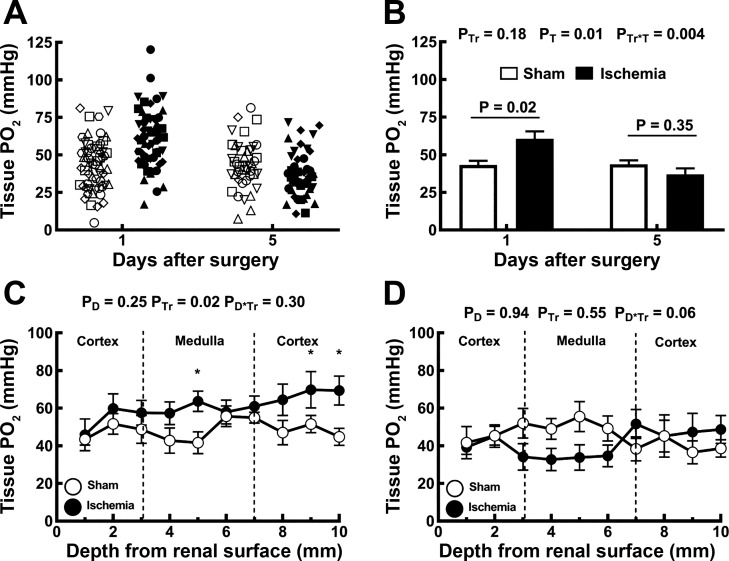
Assessment of tissue Po_2_ by Clark electrode. The electrode was first inserted 2 mm into the cortex at six random sites across the left kidney. *A*: multiple measurements are shown for each rat, with the various rats represented by different symbols. Closed symbols represent rats subjected to renal ischemia (*n* = 9 at 24 h and *n* = 8 at 5 days), whereas open symbols represent rats subjected to sham ischemia (*n* = 10 at 24 h and *n* = 7 at 5 days). *B*: measurements of cortical tissue Po_2_ for each rat were averaged and are presented as between-rat means ± SE. In *B*, *P*_Tr_, *P*_T_, and *P*_Tr*T_ are the outcomes of a two-way analysis of variance (ANOVA) with factors treatment (Tr) and time (T). *P* values above each pair of columns and error bars show the outcomes of Student’s unpaired *t*-test conservatively adjusted using the Dunn-Sidak correction with *k* = 2 to account for the fact that comparisons were made at 24 h and 5 days. *C* and *D*: a tissue Po_2_ profile with depth was established by advancing the electrode from the cortical surface at 1-mm increments, up to 10 mm into the left kidney either 24 h (*C*) or 5 days (*D*) following recovery from either ischemia or sham ischemia. Symbols and error bars are the means ± SE for rats subjected to either an hour of sham (○) or bilateral renal ischemia (●). In *C* and *D*, *P*_D_, *P*_Tr_, and *P*_D*Tr_ are the outcomes of two-way repeated-measures ANOVA with factors depth (D) and treatment. *P ≤ 0.05 and is the outcome of Student’s unpaired *t*-test without correction for multiple comparisons.

Tissue Po_2_ in the renal cortex was highly heterogenous, both 24 h and 5 days after either ischemia or sham ischemia (Fig. 2*A*). Cortical Po_2_ was, on average, 40% greater 24 h following renal ischemia than after sham ischemia. By 5 days after renal ischemia, cortical tissue Po_2_ was 39% less than 24 h after ischemia and similar to that in rats subjected to sham ischemia 5 days previously (Fig. 2*B*). Tissue Po_2_ varied little with depth from the cortical surface. At 24 h after reperfusion, tissue Po_2_ tended to be greater in rats subjected to ischemia than in those subjected to sham ischemia, the difference reaching statistical significance at depths of 5 mm (inner medulla) and 9 and 10 mm (cortex; Fig. 2*C*). Five days after renal ischemia, tissue Po_2_ did not differ significantly from its level in rats subjected to sham ischemia, at any depth below the cortical surface (Fig. 2*D*).

#### Renal hemodynamics and function.

RBF was not significantly different in rats subjected to ischemia compared with rats subjected to sham ischemia, both 24 h and 5 days after surgery (Table 2). Twenty-four hours after ischemia, mean GFR (−99%), urine flow (−82%), and sodium excretion (−85%) were less than in rats subjected to ischemia than in those subjected to sham ischemia (Table 2). Fractional excretion of sodium did not differ significantly 24 h after ischemia compared with sham ischemia. By 5 days after ischemia, renal function was highly variable between rats, with some rats having recovered relatively normal GFR while others remained in apparent renal failure. Consequently, none of these variables differed significantly from their level in rats subjected to sham ischemia. We were unable to detect a significant correlation (*r*^2^ = 0.03, *n* = 8), in rats subjected to ischemia, between GFR and tissue Po_2_ at *day 5* after surgery.

**Table 2.** Renal hemodynamic parameters of rats 24 h or 5 days after ischemia or sham ischemia

	Sham (24 h)		Ischemia (24 h)		Sham (5 days)		Ischemia (5 days)		2-Way ANOVA			Dichotomous Comparison	
Parameter	Value	*n*	Value	*n*	Value	*n*	Value	*n*	*P*_Tr_	*P*_T_	*P*_Tr*T_	S1 vs. I1	S5 vs. I5
Renal blood flow, ml/min	3.5 ± 2.9	10	3.0 ± 0.4	9	3.6 ± 0.7	7	2.4 ± 0.3	8	0.13	0.83	0.68	0.55	0.29
Renal blood flow, μl·min^−1^·g body wt^−1^	9.09 ± 0.98	10	7.93 ± 0.79	9	7.71 ± 1.59	7	6.65 ± 0.78	8	0.30	0.21	0.96	0.60	0.81
Renal plasma flow, ml/min	1.7 (1.5, 2.1)	10	1.7 (1.4, 2.0)	9	1.6 (1.5, 3.0)	7	1.6 (0.9, 1.9)	8	0.42	0.65	0.87	0.36	0.51
Renal plasma flow, μl·min^−1^·g body wt^−1^	5.1 ± 0.6	10	4.9 ± 0.5	9	4.4 ± 0.9	7	3.8 ± 0.4	8	0.77	0.31	0.94	0.96	0.80
Glomerular filtration rate, ml/min	0.8 (0.7, 1.2)	10	0.001 (0, 0.008)	9	1.0 (0.5, 1.8)	7	0.07 (0.01, 0.4)	9	<0.001	0.17	0.11	<0.001	0.14
Glomerular filtration rate, nl·min^−1^·g body wt^−1^	2,400 (1,520, 2,960)	10	3.3 (0, 2.2)	9	2,110 (1,040, 3,470)	7	190 (27, 1,080)	8	<0.001	0.18	0.03	<0.001	0.20
Urine flow, μl/min	7.0 ± 1.0	10	1.0 ± 0.5	9	10.0 ± 4.0	7	6.0 ± 2.0	8	0.01	0.09	0.99	0.001	0.36
Urine flow, nl·min^−1^·g body wt^−1^	16.0 (10.0, 28.0)	10	2.2 (0, 5.5)	9	17.0 (13.0, 25.0)	7	15 (1.1, 39.0)	8	0.04	0.05	0.06	0.002	0.66
Sodium excretion, µmol/min	0.4 (0.2, 1.0)	9	0 (0, 0.18)	9	0.20 (0.2, 0.6)	7	0.20 (0.1, 0.3)	8	0.004	0.46	0.09	0.003	0.38
Sodium excretion, nmol·min^−1^·g body wt^−1^	1.0 (0.5, 2.1)	9	0 (0, 0.47)	9	0.5 (0.36, 1.3)	7	0.6 (0.35, 0.71)	8	0.02	0.53	0.05	0.01	0.90
Sodium reabsorption, µmol/min	111.9 (93.6, 170.2)	9	0.17 (0, 1.0)	9	138.3 (63.3, 250.4)	7	8.75 (1.5, 52.0)	8	<0.001	0.2	0.15	<0.001	0.14
Sodium reabsorption, nmol·min^−1^·g body wt^−1^	307.5 (204.7, 427.6)	9	0.46 (0, 2.7)	9	310 (143.6, 479.3)	7	25.7 (3.6, 153.1)	8	<0.001	0.18	0.03	<0.001	0.20
Filtration fraction, %	46.9 (34.1, 69.8)	10	0.1 (0, 0.5)	9	48.5 (29.7, 60.5)	7	7.0 (0.6, 21.7)	8	<0.001	0.13	0.02	<0.001	0.20

Values of variables that did not violate normality are means ± SE, whereas values of variables that violated normality are expressed as medians (25th percentile, 75th percentile); *n* = no. of rats. Normality of the data was assessed using the Shapiro-Wilk test. *P*_Tr_, *P*_T_, and *P*_Tr*T_ are the outcomes of two-way analysis of variance (ANOVA) with factors treatment (Tr) and time (T) for data that did not violate normality. For data that violated normality, an ANOVA on ranking was performed instead. Dichotomous comparisons of continuous variables were made using Student’s *t*-test for data that did not violate normality. For data that violated normality, a Mann-Whitney *U*-test was performed for dichotomous comparisons. *P* values for dichotomous comparisons were conservatively adjusted using the Dunn-Sidak correction with *k* = 2 to account for the fact that comparisons were made at 24 h and 5 days. I1, 24 h after ischemia and reperfusion; I5, 5 days after ischemia and reperfusion; S1, 24 h after sham ischemia; S5, 5 days after sham ischemia.

#### Blood oximetry and renal oxygen consumption and delivery.

Arterial blood hematocrit 24 h after renal ischemia was 12% less than after sham ischemia (Table 1). We were unable to detect a significant correlation (*r*^2^ = 0.034, *n* = 9), in rats subjected to ischemia, between hematocrit and tissue Po_2_ 24 h after reperfusion. By 5 days after renal ischemia, hematocrit was similar in the two groups of rats. Arterial blood Po_2_ was 22% less, and oxygen saturation was 2.7% less, in rats 24 h after renal ischemia than after sham surgery. Renal Do_2_ tended to be (29%) less 24 h after renal ischemia than after sham ischemia, although this apparent effect was not statistically significant (*P* = 0.06). There was no significant difference in renal Do_2_ 5 days after surgery. When both time points were considered together (24 h and 5 days), renal V̇o_2_ was 55% less in rats subjected to ischemia than in those subjected to sham surgery. The fractional extraction of oxygen did not differ significantly between the treatments at either time point.

### Protocol 3: Cellular Hypoxia and Hypoxic Signaling After Renal Ischemia

#### Pimonidazole adduct immunohistochemistry.

**Fig. 3.**
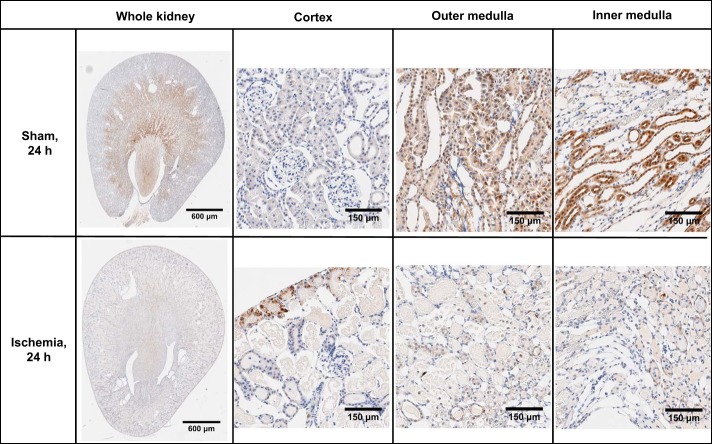
Pimonidazole adduct immunohistochemistry of renal sections 24 h following recovery from bilateral renal ischemia or sham ischemia. Images are typical of the cortical and outer and inner medullary region of the six kidneys examined in each group.

**Fig. 4.**
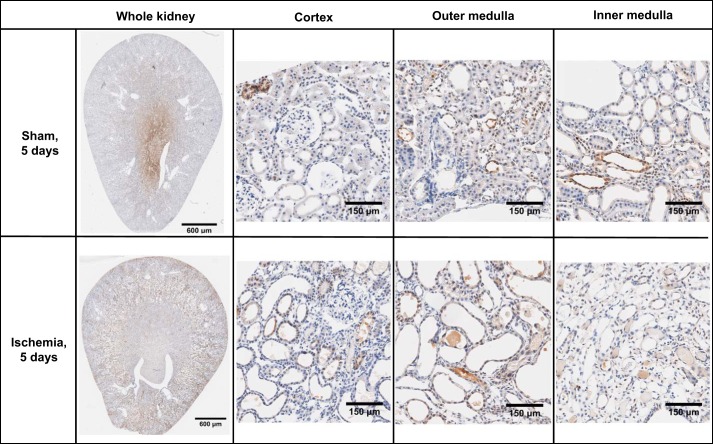
Pimonidazole adduct immunohistochemistry of renal sections 5 days following recovery from bilateral renal ischemia or sham surgery. Images are typical of the cortical and outer and inner medullary region of the six kidneys examined in each group.

No pimonidazole adducts were detected in tissues from rats that did not receive pimonidazole chloride or in sections that were not incubated with the primary antibody (data not shown). Kidney sections from sham-operated rats appeared morphologically normal (Figs. 3 and 4). Pimonidazole adducts were largely absent in the cortical region of rats 24 h following sham ischemia. However, there was diffuse staining of pimonidazole adducts in tubular elements of the outer and inner medulla following sham ischemia. Kidney sections from rats 24 h following recovery from renal ischemia showed relatively little staining for pimonidazole adducts across all regions of the kidney, but some diffuse staining was present 5 days following ischemia and reperfusion. However, luminal aspects of tubules were often stained positive for pimonidazole adducts after renal ischemia, suggestive of marked tubular obstruction. There was significant cellular sloughing and disintegration of the brush border/apical membrane of tubules after renal ischemia. In addition, there were considerable cellular debris in the luminal aspects of tubules at 24 h after renal ischemia. Tubular profiles surrounding the debris-riddled tubules were often flattened. In contrast, tubules appeared to be mostly dilated 5 days after renal ischemia. By 5 days after ischemia, tubules in the cortex and outer and inner medulla appeared to be more dilated than after sham ischemia or 24 h after renal ischemia.

#### HIF-1α and HIF-2α proteins.

**Fig. 5.**
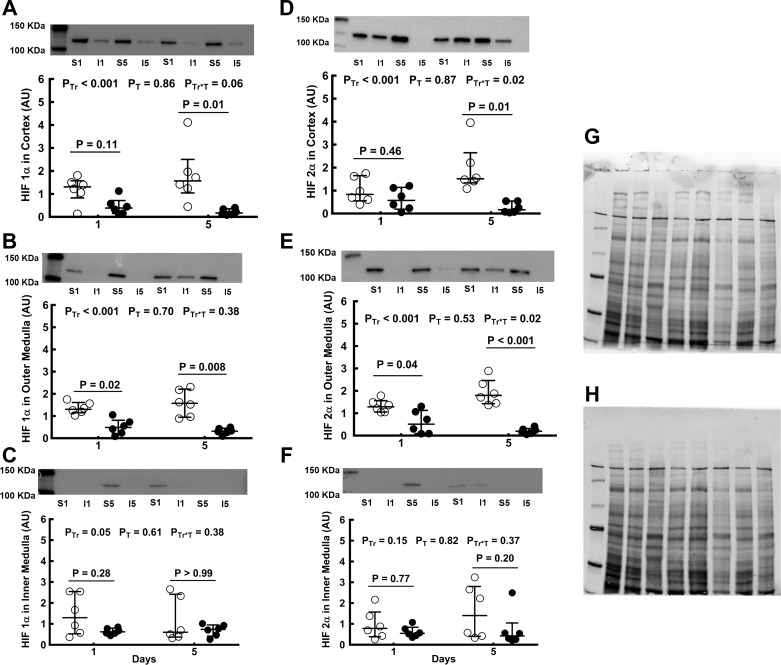
Expression of hypoxia-inducible factor (HIF) proteins after bilateral renal ischemia or sham ischemia. Immunoblots for HIF-1α (*A*–*C*) and HIF-2α (*D*–*F*) of tissue extracts from the cortex and outer and inner medulla of the left kidneys of rats 24 h and 5 days following recovery from either sham ischemia (○) or bilateral renal ischemia (●); *n* = 6 per group. *G*: typical image of the gel following electrophoresis. *H*: typical image of the nitrocellulose membrane following transfer. Values are expressed as medians (25th percentile, 75th percentile). Paired comparisons were performed using the Mann-Whitney *U*-test. Because paired comparisons were made at two time points, *P* values were conservatively adjusted using the Dunn-Sidak method with *k* = 2. *P*_Tr_, *P*_T_, and *P*_Tr*T_ are the outcomes of two-way analysis of variance on ranking with the factors treatment (Tr) and time (T). AU, arbitrary unit; I1, 24 h after ischemia; I5, 5 days after ischemia; S1, 24 h after sham ischemia; S5, 5 days after sham ischemia.

When both the 24-h and 5-day time points were considered collectively, the expression of HIF-1α protein in the cortex, outer medulla, and inner medulla was less after renal ischemia than after sham ischemia (Fig. 5, *A*–*C*). However, not all comparisons at individual time points were statistically significant. HIF-1α levels in the cortex were 88.3% less 5 days after renal ischemia than at the corresponding time-point after sham ischemia (Fig. 5*A*). Similarly, in the outer medulla, HIF-1α was 62.2% less 24 h after renal ischemia and 79.7% less 5 days after renal ischemia than after sham surgery (Fig. 5*B*). In contrast, in the inner medulla, levels of HIF-1α protein did not differ significantly, between rats subjected to ischemia and those subjected to sham ischemia, at either the 24-h or 5-day time point (Fig. 5*C*). When both the 24-h and 5-day time points were considered collectively, the expression of HIF-2α protein was markedly less, in rats subjected to ischemia compared with those subjected to sham ischemia, in the cortex and the outer medulla but not in the inner medulla. The level of HIF-2α in the cortex was 86.9% less 5 days after ischemia than after sham ischemia (Fig. 5*D*). In the outer medulla of rats subjected to renal ischemia, HIF-2α expression was 55% less 24 h and 89.2% less 5 days after ischemia than after sham ischemia (Fig. 5*E*). The deficits in HIF-1α and HIF-2α in rats subjected to renal ischemia did not diminish between the 24-h and 5-day time points, if anything, becoming more marked (Fig. 5).

#### Expression of genes for HIF-1α, HIF-2α, VEGF-α, and HO-1.

**Fig. 6.**
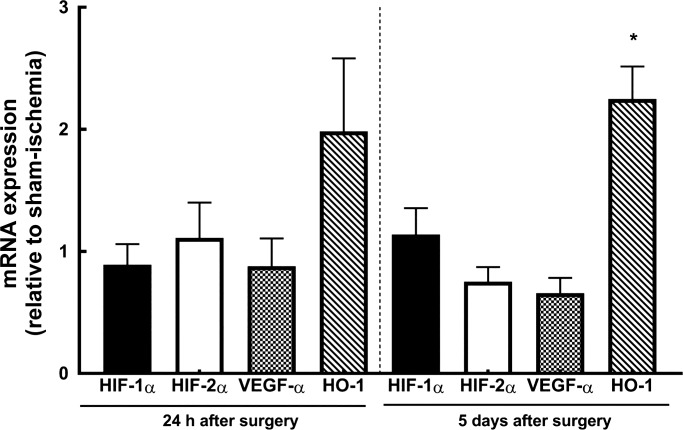
mRNA expression of HIF-1α, HIF-2α, VEGF-α, and heme oxygenase 1 (HO-1). Expression of HIF-1α, HIF-2α, VEGF-α, and HO-1 mRNA is presented as relative to that of control animals. Values are expressed as means ± SE. *P ≤ 0.05 for specific comparisons between the two treatment groups at each time point using Student’s unpaired *t*-test.

There were no significant differences in the expression of mRNA for HIF-1α, HIF-2α, or VEGF-α, either 24 h or 5 days following renal ischemia compared with after sham ischemia (Fig. 6). The expression of HO-1 mRNA tended to be greater after ischemia than after sham ischemia, although this apparent effect was only statistically significant at the 5-day time point.

#### Collagen deposition.

**Fig. 7.**
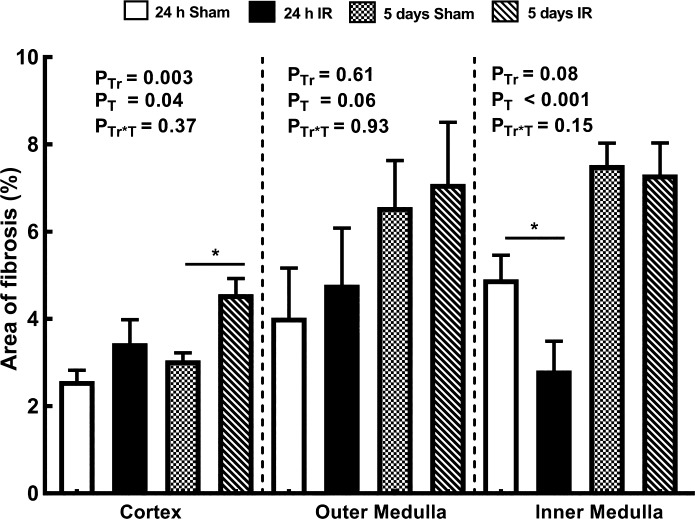
Collagen deposition in kidneys of rats. The percent areas of interstitial fibrosis relative to the areas of the cortex and outer and inner medulla are shown for rats 24 h and 5 days after recovery from either sham ischemia or bilateral renal ischemia (IR); *n* = 6 per group. Values are expressed as means ± SE. Paired comparisons were performed using Student’s unpaired *t*-test (*P ≤ 0.05). Because paired comparisons were made at two time points, *P* values were conservatively adjusted using the Dunn-Sidak method with *k* = 2. *P*_Tr_, *P*_T_, and *P*_Tr*T_ are the outcomes of two-way analysis of variance with the factors treatment (Tr) and time (T).

Twenty-four hours after renal ischemia, picrosirius red staining did not differ significantly from that seen in rats subjected to sham ischemia in either the cortex or the outer medulla. However, it was 43% less in the inner medulla (Fig. 7). By 5 days after renal ischemia, picrosirius red staining was 50% greater in the cortex of rats subjected to ischemia than in those subjected to sham ischemia. There was an apparent effect of the duration of recovery period on picrosirius red staining, which in the cortex and inner medulla was significantly greater 5 days after ischemia or sham ischemia than at the 24-h time point.

#### Indexes of renal dysfunction.

**Fig. 8.**
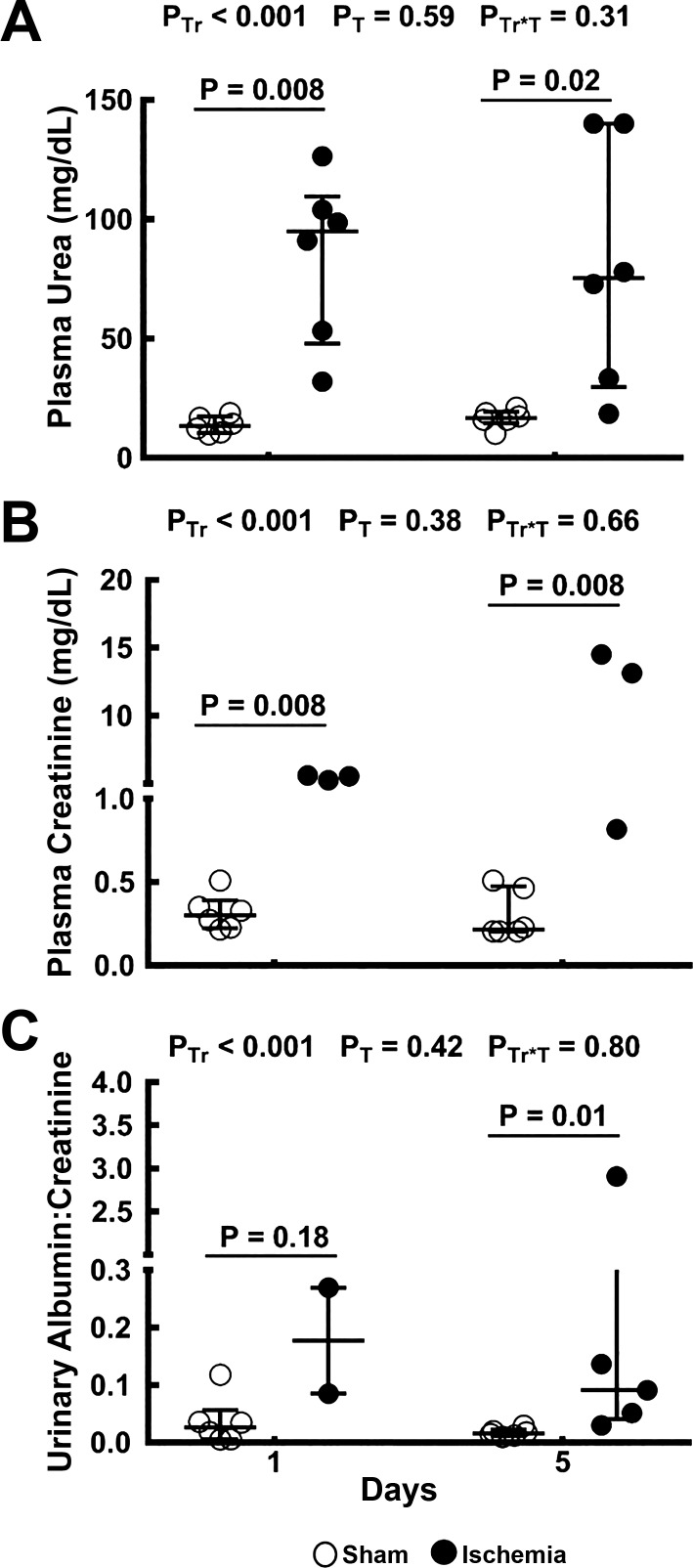
Indicators of renal dysfunction. Plasma concentrations of urea (*A*) and creatinine (*B*) and the urinary albumin-to-creatinine ratio (*C*) are shown for rats 24 h and 5 days after sham ischemia (○) or bilateral renal ischemia (●); *n* = 6 per group. Values are expressed as medians (25th percentile, 75th percentile). Comparisons were performed using the Mann-Whitney *U*-test. Because comparisons were made at two time points, *P* values were conservatively adjusted using the Dunn-Sidak method with *k* = 2. *P*_Tr_, *P*_T_, and *P*_Tr*T_ are the outcomes of two-way analysis of variance on ranking with the factors treatment (Tr) and time (T).

The plasma concentrations of urea and creatinine and the urinary albumin-to-creatinine ratio were all greater in rats after ischemia than after sham ischemia (Fig. 8). These effects were statistically significant at the individual time points with the exception of the urinary albumin-to-creatinine ratio 24 h after ischemia, where sufficient urine for analysis could only be generated from two animals.

## DISCUSSION

We determined the time course of changes in, and the spatial distribution of, renal tissue Po_2_ during the subacute phase of severe renal IRI. Using four different methods for assessing renal tissue oxygenation, we could not detect tissue hypoxia during the extension/recovery phase of IRI. Indeed, if anything, there was relative hyperoxia up to 48 h after an hour of bilateral renal ischemia. We also observed downregulation of the abundance of HIF-1α and HIF-2α protein, particularly in the cortex and outer medulla, both 24 h and 5 days after reperfusion. The apparent absence of renal hypoxia is consistent with the pattern of changes in renal Do_2_ and V̇o_2_ after ischemia and reperfusion. That is, RBF was relatively normal, but there was a marked reduction in sodium reabsorption, and so presumably oxygen utilization for sodium reabsorption, at both 24 h and 5 days after reperfusion. When both time points were considered together, renal V̇o_2_ was significantly less, and Do_2_ tended to be less, in rats subjected to ischemia than in those subjected to sham ischemia. Thus, tissue Po_2_ appears to be well maintained during the extension/recovery phase of severe renal IRI because changes in renal Do_2_ and V̇o_2_ are relatively balanced.

The methods we used to assess renal oxygenation have both strengths and weaknesses (11, 33). Radiotelemetry allows continuous measurement of renal tissue Po_2_ in the absence of confounding effects of anesthesia (22, 23). However, tissue Po_2_ can only be expressed in relative terms and can be measured at only one site in each animal. Clark electrodes allow generation of a spatial map of tissue Po_2_, but only in anesthetized animals (11, 33). Furthermore, it is not possible to resolve tissue Po_2_ to the level of specific vascular and tubular elements, except in the superficial cortex (43). In addition, as we have found previously with Clark electrodes inserted into renal tissue from the dorsal surface of the kidney (32), the steep corticomedullary gradient in tissue Po_2_ generated in many previous studies (6, 10, 29) is not obviously evident. We have no adequate explanation for this, although it may relate to our use of relatively large electrodes (50 μm) or the angle of entry to the renal tissue, from the dorsal surface of the kidney, as a consequence of which the tip of the electrode does not enter the renal papilla. Pimonidazole adduct immunohistochemistry allows detection of cells with Po_2_ < 10 mmHg but does not provide a quantitative measure of tissue Po_2_ (37). Furthermore, as we found in the present study and previously (1), it is prone to artifactual staining of cellular debris and casts within damaged tubules. Quantification of the abundance of HIF-1α and HIF-2α protein provides information about the state of hypoxia signaling pathways. However, factors other than tissue Po_2_ contribute to the regulation of HIF signaling (16). Thus, interpretation of our failure to detect hypoxia by any one of these methods would merit caution. However, the fact that our observations were consistent across the four methods provides compelling evidence that at least in this severe form of IRI, tissue hypoxia is not an obligatory characteristic of the period from 24 h to 5 days after severe renal ischemia and reperfusion.

The most likely explanation for the absence of hypoxia 24 h and 5 days after reperfusion, and even increased tissue Po_2_ at 24 h, is reduced sodium reabsorption and thus renal V̇o_2_. In the rats we studied, the deficit in sodium reabsorption 24 h after ischemia and reperfusion could be attributed to the decreased filtered load of sodium. This appears to drive downregulation of Na^+^-K^+^-ATPase activity. For example, in response to severe renal ischemia (i.e., 60 min), the abundance (and activity) of basolateral Na^+^-K^+^-ATPase pumps and the apical Na-K-2Cl and thiazide-sensitive Na^+^-Cl^−^ cotransporters were shown to be greatly reduced (25). However, the magnitude of the apparent reduction in renal V̇o_2_ we observed was considerably less than the magnitude of the reduction in sodium reabsorption. For example, sodium reabsorption was <1% of rats subjected to sham ischemia, whereas V̇o_2_ was 34% than that of rats subjected to sham ischemia 24 h after reperfusion. These observations are consistent with the concept that oxygen utilization for sodium reabsorption becomes less efficient in AKI. In support of this concept, Redfors et al. studied renal oxygen utilization in patients with AKI subsequent to cardiothoracic surgery (35). They found a deficit in sodium reabsorption of 59% in patients with AKI after cardiothoracic surgery compared with patients without AKI (35). In contrast, renal V̇o_2_ was similar in the two groups of patients. Furthermore, renal V̇o_2_ per unit of reabsorbed sodium was 2.4 times greater in patients with AKI than in those without AKI (35). The inefficiency of oxygen utilization for sodium reabsorption in AKI appears to be driven by multiple factors, including loss of polarity of Na^+^-K^+^-ATPase pumps, oxidative stress, and reduced bioavailability of nitric oxide (17, 24).

Renal tissue Po_2_ is determined by the balance between local Do_2_ and V̇o_2_ (12). Thus, tissue Po_2_ during recovery from AKI is likely to be model dependent. In a model of severe AKI such as the one used in the present study, in which the filtered load of sodium (and thus oxygen utilization for sodium reabsorption) is greatly reduced but RBF (and thus presumably local tissue Do_2_) is well preserved, the absence of tissue hypoxia, and even tissue hyperoxia, might be expected. On the other hand, tissue hypoxia might be predicted in a model of less severe renal dysfunction, and thus better preserved GFR. This concept is consistent with clinical observations in patients after renal transplantation. Using blood oxygen level-dependent magnetic resonance imaging, Sadowski et al. observed greater renal medullary oxygenation in the transplanted kidneys of patients with acute allograft rejection than in patients with normal functioning allografts, despite the former having a deficit in renal medullary perfusion (38). Similarly, Rosenberger et al. observed low HIF-1α abundance in biopsies of patients with nonfunctional allografts but induction of HIF-1α in biopsies from functional grafts (36). Thus, there is a strong rationale for the methods used in the present study to be applied to a less severe model of AKI, in which tissue hypoxia might be more likely to occur.

It is noteworthy that HIF-1α and HIF-2α protein expression was downregulated not just at 24 h after reperfusion, presumably driven in part by increased tissue oxygen availability, but also 5 days after reperfusion, when tissue Po_2_ was similar in rats exposed to ischemia and sham ischemia. Inhibition of HIF-1α and HIF-2α abundance appears to be mediated by posttranslational processes at both 24 h and 5 days after reperfusion, since the expression of mRNA for these proteins was relatively normal at both time points. The bioavailability of HIFs is influenced by various factors, such as their phosphorylation (20) and hydroxylation of proline and asparagine residues on HIFs (44) that target these protein for ubiquitinylation. The levels of proline hydroxylases (PHDs) have been shown to be unaltered following ischemia and reperfusion of the kidney (13, 39). A caveat to that is that the posttranslational modification of HIFs by PHDs in the kidney is likely complex given that the expression patterns, and thus sensitivity, of PHDs vary in different regions of the kidney likely because of the heterogeneity of renal tissue Po_2_ under physiological conditions (39). It is also noteworthy that mRNA for VEGF-α and HO-1, genes under the control of the HIF-1α and HIF-2α promoter, were not downregulated at 24 h or 5 days after reperfusion. This observation is consistent with the concept that factors other than HIFs regulate expression of these genes in the subacute phase of severe IRI. The signaling pathway for VEGFs is complex and is critical for neovascularization. A myriad of factors apart from HIFs, such as VEGF receptor signaling complexes and neurolipin, are able to modulate the abundance and activity of VEGFs (21). Kanellis et al. showed that expression of VEGF was unaltered in response to ischemia-reperfusion of the kidney (18). Interestingly, the expression of VEGF receptor 2 was increased following ischemia, and VEGF was redistributed to the basolateral membrane, consistent with the established role of VEGF in the maintenance of an adequate blood supply, in remaining viable tissues, as evinced in the present study by relatively well-maintained RBF (19). Nevertheless, the permanent loss of peritubular capillaries, due to inadequate vascular reparation and/or neovascularization, appears to be an important event in the progression from ischemia-induced AKI to CKD (2, 4).

### Perspectives and Significance

In models of AKI induced by complete renal ischemia, hypoxia during the period of ischemia is obligatory and is likely one of the drivers of necrosis and apoptosis associated with the development of AKI after reperfusion. Furthermore, other important factors during reperfusion, such as oxidative stress (5, 7) and influx of immune-modulatory cells (14, 42), are initiated, at least partly, by the hypoxia during ischemia. In the first few hours after reperfusion (acute phase), reduced renal tissue or microvascular Po_2_ has been observed in some (27, 28), but not all (1), cases. To the best of our knowledge, our present report describes the first detailed investigation of tissue oxygenation during the subacute phase of renal IRI. We provide compelling evidence that at least in severe IRI modeling subacute and end-stage renal disease, renal tissue hypoxia is not present 24 h and 5 days after reperfusion. It is possible that the absence of hypoxia at these time points in this experimental model of severe IRI is a consequence of the degree of renal damage and the consequent deficit in renal oxygen consumption. Thus, future studies should focus on less severe models of AKI and follow animals for longer periods after reperfusion, to better characterize the natural history of renal oxygenation during progression from AKI to CKD.

## GRANTS

This work was supported by National Health and Medical Research Council of Australia Grants GNT606601 and GNT1024575. M. P. Koeners is supported by the British Heart Foundation (FS/14/30630) and the European Union, Seventh Framework Programme, Marie Curie Actions [Cardio Renal Paradigms Elucidated through an International Exchange Scheme (CARPEDIEM) Grant 612280 and International Outgoing Fellowship 282821]. M. J. Watt is supported by National Health and Medical Research Council of Australia Grant GNT1077703.

## DISCLOSURES

No conflicts of interest, financial or otherwise, are declared by the authors.

## AUTHOR CONTRIBUTIONS

C.P.C.O. and R.G.E. conceived and designed research; C.P.C.O., J.P.N., and G.B. performed experiments; C.P.C.O. analyzed data; C.P.C.O., J.P.N., and R.G.E. interpreted results of experiments; C.P.C.O. prepared figures; C.P.C.O. drafted manuscript; C.P.C.O., J.P.N., M.M.U., G.B., R.C.M., M.J.W., L.M.H., M.P.K., and R.G.E. edited and revised manuscript; C.P.C.O., J.P.N., M.M.U., G.B., R.C.M., M.J.W., L.M.H., M.P.K., and R.G.E. approved final version of manuscript.
